# Human Recombinant Fab Fragment Neutralizes Shiga Toxin Type 2 Cytotoxic Effects *in vitro* and *in vivo*

**DOI:** 10.3390/toxins10120508

**Published:** 2018-12-02

**Authors:** Daniela Luz, Maria Marta Amaral, Flavia Sacerdoti, Alan Mauro Bernal, Wagner Quintilio, Ana Maria Moro, Marina Sandra Palermo, Cristina Ibarra, Roxane Maria Fontes Piazza

**Affiliations:** 1 Laboratório de Bacteriologia, Instituto Butantan, São Paulo 05503900, Brasil; 2 Laboratorio de Fisiopatogenia, Departamento de Fisiología, Instituto de Fisiología y Biofísica Bernardo Houssay (IFIBIO Houssay-CONICET), Facultad de Medicina, Universidad de Buenos Aires, Buenos Aires C1121, Argentina; mmamaral74@gmail.com (M.M.A.); flasacerdoti@gmail.com (F.S.); cristinaadrianaibarra@gmail.com (C.I.); 3 Laboratorio de Patogénesis e Inmunología de Procesos Infecciosos, Instituto de Medicina Experimental, (IMEX)-CONICET-Academia Nacional de Medicina, Buenos Aires C1425, Argentina; alanmbernal@gmail.com (A.M.B.); marinasandrapalermo@gmail.com (M.S.P.); 4 Laboratório de Biofármacos em Células Animais, Instituto Butantan, São Paulo, SP 05503-900, Brazil; wagner.quintilio@butantan.gov.br (W.Q.); ana.moro@butantan.gov.br (A.M.M.)

**Keywords:** Shiga toxin, recombinant antibody fragment, Fab, neutralization, protection

## Abstract

Shiga toxin (Stx) producing *Escherichia coli* (STEC) is responsible for causing hemolytic uremic syndrome (HUS), a life-threatening thrombotic microangiopathy characterized by thrombocytopenia, hemolytic anemia, and acute renal failure after bacterially induced hemorrhagic diarrhea. Until now, there has been neither an effective treatment nor method of prevention for the deleterious effects caused by Stx intoxication. Antibodies are well recognized as affinity components of therapeutic drugs; thus, a previously obtained recombinant human FabC11:Stx2 fragment was used to neutralize Stx2 in vitro in a Vero cell viability assay. Herein, we demonstrated that this fragment neutralized, in a dose-dependent manner, the cytotoxic effects of Stx2 on human glomerular endothelial cells, on human proximal tubular epithelial cells, and prevented the morphological alterations induced by Stx2. FabC11:Stx2 protected mice from a lethal dose of Stx2 by toxin-antibody pre-incubation. Altogether, our results show the ability of a new encouraging molecule to prevent Stx-intoxication symptoms during STEC infection.

## 1. Introduction

Shiga toxin type 2 (Stx2) is a ribotoxin produced by several strains of Shiga toxin-producing *Escherichia coli* (STEC), an etiologic agent of bloody diarrhea. Stx2 consists of seven different variants (Stx2a–Stx2g), which have high homology (93–100%), except for Stx2f (69% identity) [1]. These toxins are encoded by a bacteriophage genome, which can be transferred between related bacteria, resulting in a diverse array of bacterial strains secreting one or more toxin subtypes [2].

Stx2 is part of a related bacterial toxin protein family that are similar in structure and mechanism of action, comprised of Stx from *Shigella dysenteriae* and Stx1 from STEC. Stxs belong to the AB_5_ toxin protein class, consisting of an enzymatically active A subunit (~32 kDa), and a homo-pentameric B subunit (7.7 kDa per monomer) which binds to the host receptor globotriaosylceramide (Gb_3_) [3]. After receptor-mediated endocytosis, the Stx enzymatically active A subunit depurinates the conserved adenine residue of 28S eukaryotic rRNA, terminating peptide elongation and leading to cell death [4]. Stx2-producing strains are more virulent than Stx1-producing ones and are frequently associated with hemolytic uremic syndrome (HUS), a thrombotic microangiopathy characterized by thrombocytopenia, hemolytic anemia, and acute renal failure [5]. This syndrome can lead to death or long-term consequences such as hypertension and renal disease, because microvascular endothelial cells in the kidney are highly sensitive to Stx [6].

Until now, there is neither an effective treatment nor prevention method for the deleterious effects of Stx intoxication [3]. However, several strategies have been developed, such as controlling bacterial growth without increasing Stx secretion, interference with toxin trafficking and cellular response to the toxin. Designing antibodies against Stx is an important challenge for HUS therapy [7]. Using antibodies directed against Stx as therapy either to prevent or treat the HUS disease process is a promising approach [8,9,10]. Since human microvascular endothelial cells express 50-fold higher Gb_3_ levels compared to the endothelial cells of large vessels [11], they are an excellent cell model for *in vitro* therapeutic studies.

Antibodies are well recognized as affinity reagents and therapeutic drugs; thus, they can be generated with high affinity and specificity. They are also long lived in circulation and are generally well tolerated as drugs [12]. Phage display antibody libraries provide an attractive substitute to circumvent animal immunization and other caveats of hybridoma technology, an effective but laborious and expensive approach to generate monoclonal antibodies [12].

A human recombinant monoclonal Fab fragment against Stx2 (FabC11:Stx2) was previously obtained by phage display technology [13] using a synthetic library [14]. This fragment was produced in a bacterial system, which is more feasible and economical than conventional hybridoma technology, and showed a detection limit of 24 nM to detect Stx2 and cross-reacted to Stx1, which was confirmed by peptide array, showing that this molecule recognizes the B subunit of both toxins, although with higher affinity to Stx2B. This fragment also demonstrated neutralizing ability against Stx2 in Vero cell assay. Altogether, these features suggest that this antibody fragment might be an alternative therapy against Stx2 intoxication symptoms. Herein, the affinity and ability of this fragment, which neutralize the toxicity of Stx2, were further evaluated by testing two different human renal cell lines and the fragment’s ability to protect mice from Stx2 intoxication. 

## 2. Results

### 2.1. FabC11:Stx2 Has High Affinity to Stx2 and Protects Human Glomerular Endothelial Cells (HGEC) and Human Kidney (HK-2) Cells from Stx2 Cytotoxicity

First, the kinetic affinity constant (K*_D_*) of the fragment to Stx2 was determined using Surface Plasma Resonance (SPR), since previous work had showed only the apparent affinity [13]. Using this accurate methodology, the K*_D_* was found to be 7.54 × 10^−9^ M (Table 1).

**Figure 1:**
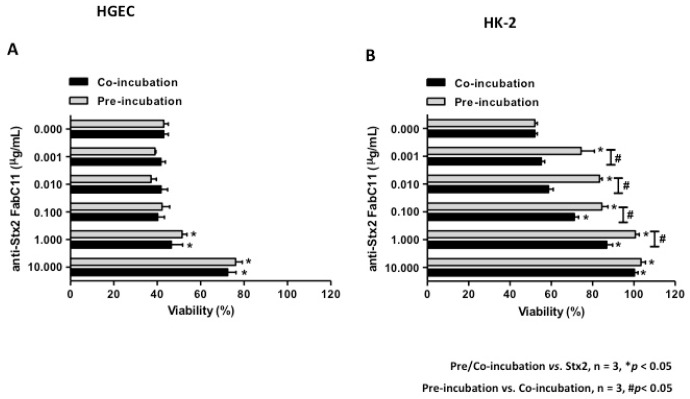
FabC11:Stx2 protected HGEC and HK-2 against Stx2 cytotoxicity. HGEC (**A**) and HK-2 (**B**) were pre-treated with different concentrations of FabC11:Stx2 (1 h at 37 °C), and Stx2 (0.1 ng/mL) was then added, or cells were co-treated with FabC11:Stx2 (0.001 µg/mL to 10 µg/mL) and Stx2 (0.1 ng/mL) simultaneously. After 72 h of treatment, cells were incubated with neutral red for an additional 1 h at 37°C in 5% CO_2_. Absorbance of each well was read at 540 nm. One hundred percent represents cells incubated under identical conditions but without FabC11:Stx2 or toxin treatment (Ctrl). Results are expressed as means ± SEM of three experiments, pre/co-incubation vs. Stx2, * *p* < 0.05; pre-incubation vs. co-incubation, # *p* < 0.05.

To analyze the capacity of FabC11:Stx2 to neutralize Stx2 cytotoxicity, we developed HGEC and HK-2 viability assays by neutral red uptake assay. Cells were pre-treated with FabC11:Stx2 (0.001 to 10 µg/mL) for 1 h at 37 °C and then exposed to Stx2 (0.1 ng/mL) for 72 h, or cells were co-incubated simultaneously with FabC11:Stx2 (0.001 to 10 µg/mL) and Stx2 (0.1 ng/mL) and incubated for 72 h. The molar ratios used in these experiments were from 0.7:1 to 7000:1 for Fab:Stx2. Under both conditions evaluated, pre-incubation and co-incubation, FabC11:Stx2 significantly neutralized the cytotoxic effects of Stx2 on HGEC and HK-2 cells (*p* < 0.05, *n* = 3). These effects were shown to be dose-dependent. For HGEC, the highest protection of FabC11:Stx2 was observed at 10 µg/mL. No significant differences were found between the two experimental conditions, since cell viability with 10 µg/mL FabC11:Stx2 was 54 ± 3.0% and 52 ± 4.0% under pre-incubation and co-incubation conditions, respectively (Figure 1A).

In addition, the loss of HK-2 viability was significantly inhibited with lower FabC11:Stx2 concentrations (0.001 µg/mL), compared to HGEC (1 µg/mL) (Figure 1B). Furthermore, pre-incubation with 0.001–1 µg/mL FabC11:Stx2 was more effective than co-incubation (FabC11:Stx2 1 µg/mL) in neutralizing Stx2 cytotoxicity (100 ± 2.0% vs. 82.0 ± 3.0%). The neutralization activity of Stx2 in HK-2 was 100% with 1 µg/mL and 10 µg/mL FabC11:Stx2 for pre-incubation and co-incubation, respectively.

### 2.2. FabC11:Stx2 Prevents Morphological Alterations Induced by Stx2 in HGEC and HK-2 Cells

**Figure 2:**
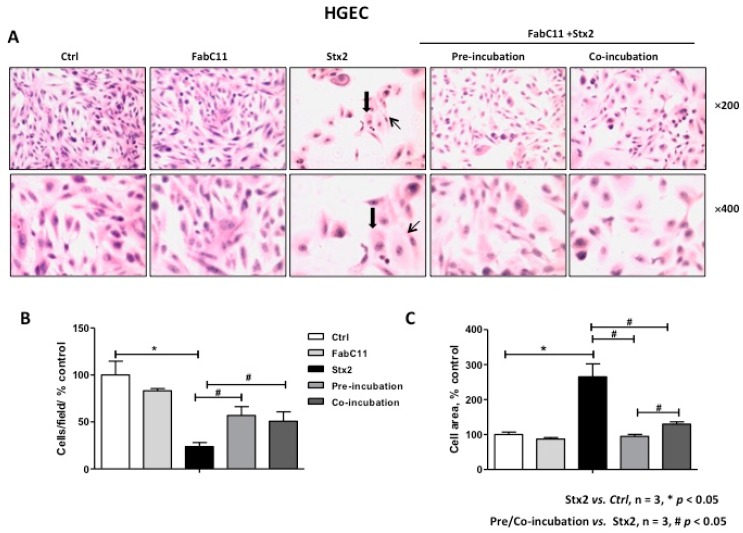
FabC11:Stx2 protects HGEC from Stx2-induced morphological disturbances. HGEC seeded on gelatin-coated glass coverslips was pre-treated with FabC11:Stx2 (10 µg/mL, 1 h at 37 °C), and Stx2 (0.1 ng/mL) was then added. Co-incubation was performed by adding FabC11:Stx2 (10 µg/mL) with Stx2 (0.1 ng/mL) simultaneously. After 72 h of treatments, cells were stained with Hematoxylin and Eosin (H&E) and the morphology (**A**) and number of HGEC (**B**) were analyzed by light microscopy (×200 and ×400). HGEC areas (**C**) were measured using ImageJ software. The black arrows indicate the morphological changes such as intracellular edema (thick arrows) and elongated shape (thin arrows). Results are expressed as means ± SEM of three experiments. One hundred percent represents values of controls. Stx2 vs. Ctrl, * *p* < 0.05. Pre/co-incubation vs. Stx2, # *p* < 0.05.

**Figure 3:**
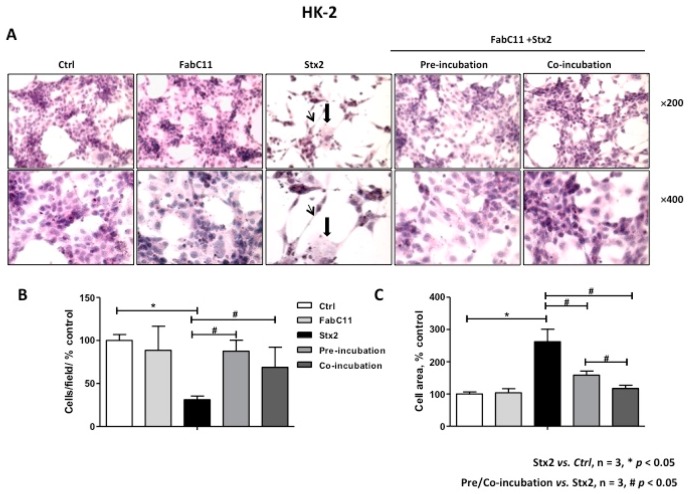
FabC11:Stx2 protects HK-2 from Stx2-induced morphological disturbances. HK-2 seeded on glass coverslips was pre-treated with FabC11:Stx2 (10 µg/mL, 1 h at 37 °C), and Stx2 (0.1 ng/mL) was then added. Co-incubation was performed by adding FabC11:Stx2 (10 µg/mL) with Stx2 (0.1 ng/mL) simultaneously and incubating cells for 72 h. Finally, cells were stained with H&E, and the morphology (**A**) and number of HK-2 (**B**) were analyzed by light microscopy (×200 and ×400). HK-2 areas (**C**) were measured using ImageJ software. The black arrows indicate the morphological changes, namely intracellular edema (thick arrows) and elongated shape (thin arrows). Results are expressed as means ± SEM of three experiments. One hundred percent represents values of controls. Stx2 vs. Ctrl, * *p* < 0.05. Pre/co-incubation vs. Stx2, # *p* < 0.05.

As previously described, Stx2 causes morphological alterations associated with cell detachment and intracellular edema on HGEC and HK-2 cells [7,16]. Therefore, the morphology of HGEC and HK-2 cells treated with Stx2 and in the presence of FabC11:Stx2 antibodies was analyzed. The results showed that this recombinant antibody fragment significantly decreased cell detachment and intracellular edema caused by Stx2 in HGEC (Figure 2) and HK-2 cells (Figure 3). No significant differences were found for prevention of HGEC or HK-2 cell detachment between pre-incubation and co-incubation conditions (Figure 2B and Figure 3B ). However, prevention of HGEC intracellular edema was higher with pre-incubation than co-incubation (Figure 2C), and, on the contrary, prevention of HK-2 intracellular edema was higher with co-incubation than pre-incubation (Figure 3C).

### 2.3. FabC11:Stx2 Protected Mice Injected with a Lethal Dose of Stx2 

**Figure 4:**
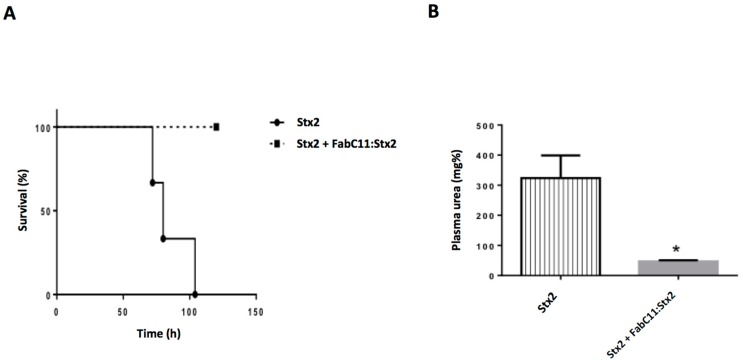
In vivo neutralizing activity of FabC11:Stx2. (**A**) In vivo protection against a lethal challenge with recombinant Stx2 (rStx2). Six adult BALB/c mice were challenged with an intravenous (i.v.) injection of 1 LD_100_ of Stx2 pre-incubated with PBS (Stx2) or FabC11:Stx2 (FabC11:Stx2), as detailed in Materials and Methods. The two survival curves were statistically different by the log-rank (Mantel–Cox) test (*p* < 0.02). (**B**) Plasma urea nitrogen levels (mg %) were determined at 72 h after Stx2 intoxication. Each bar represents the mean ± SEM of 3 mice from each group. * *p* < 0.02 vs. Stx2 intoxicated mice by unpaired *t*-test.

To further analyze the protective capacity of FabC11:Stx2, we challenged adult mice with a lethal dose (1 LD_100_) of rStx2. The molar ratio used in this experiment was 2 × 10^6^: 1 Fab:Stx2, which means 144 µg of Fab/mouse. Figure 4A shows that 100% of mice injected with 1 LD_100_ Stx2 pre-incubated with FabC11:Stx2 survived, compared to mice injected with rStx2 pre-incubated in PBS (0% survival). 

Plasma urea levels were measured in these mice as a functional parameter of kidney damage and indicator of Stx2 toxicity. As seen in Figure 4B, mice injected with 1 LD_100_ of rStx2 in the presence of FabC11:Stx2 showed normal urea levels. In contrast, high urea levels were observed in Stx2-injected mice, demonstrating the efficacy of FabC11:Stx2 against Stx2 toxicity.

## 3. Discussion

HUS is the major and more complicated pathology related to STEC infections, leading to death or severe sequelae, in which Stx toxins are the main STEC virulence factors. Consequently, its neutralization is a critical therapeutic approach. Since the first STEC outbreak in 1982, several studies have been dedicated to Stx neutralization strategies and HUS prevention. However, to date, to treat or prevent HUS, only a few molecules have reached trials phase I and II. 

Indeed, SYNSORB Pk, a receptor analogue which in theory binds to free Stx in the infected patient’s gut, prevents the toxin from acting either locally or systemically [17]. The antibody urtoxazumab (TMA-15), a humanized monoclonal antibody against the Stx2 B subunit was well tolerated in phase I studies [18] and reached phase II, but data on efficacy has not yet been published. The humanized antibodies cαStx1 and cαStx2 against both Stx1 and Stx2 B subunits were also shown to be well tolerated in phase I studies [19] and are now in phase II trials [10]. In summary, the few promising molecules for preventing Stx effects and HUS development are still clinically unavailable because they are still under investigation, or because studies were inconclusive for their therapeutic use. Thus, at present, there is no treatment to prevent the occurrence of HUS and other complications caused by STEC.

Although monoclonal antibodies (mAb), a growing category of pharmaceutical proteins, are generated dominantly by hybridoma technology, advances in *in vitro* selection technology, such as phage display, allows mAb generation with less time and cost compared to hybridoma procedures [20]. With this technique, human antibodies can be obtained directly without needing the cumbersome humanization of murine antibodies. Furthermore, some synthetic libraries consist of a highly stable framework, which minimizes immunogenicity, ideal for a therapeutic molecule [21]. Moreover, some bacterial systems for recombinant expression and purification, such as FabC11:Stx2 [13], are lower-cost alternatives for production of these antibodies [22].

It has been shown that Stx2 is more lethal than Stx1 in animal models, and it is also the main cause of severe outcomes of STEC infections in humans and HUS development [3]. The FabC11:Stx2 antibody binds to the Stx2 B subunit with high affinity (7.54 × 10^−9^ M) and cross-reacts with Stx1; additionally, it is capable of neutralizing Stx2 in vitro. In addition, here we demonstrated that FabC11:Stx2 protected against loss of cell viability and altered morphology of HGEC primary culture and the HK-2 cell line due to Stx2 cytotoxicity. We found that HK-2 cells were protected by lower concentrations of FabC11:Stx2 than HGEC, and pre-incubation treatment was more effective than co-incubation. On the contrary, HGEC cell protection was only obtained at the highest concentrations of FabC11:Stx2; assayed and pre-incubation and co-incubation conditions gave the same protection.

In diarrhea-associated HUS, kidneys are the organs predominantly damaged by Stx, because of the existence of microvascular endothelial cells, which express high levels of Gb_3_ [11]. We have previously shown that HGEC and HK-2 cells exhibited the same sensibility to Stx2 [7]. Taking this into account, the differences between the protection of HGEC and HK-2 could be due to a possible competition effect between Gb_3_ receptor and FabC11:Stx2 antibody for binding Stx2, or because of a different availability of FabC11:Stx2 antibodies in the cellular environment. In this sense, endocytosis of Fab fragments by endothelial cells have been previously described [23], so the concentration of antibodies to neutralize the toxin could decrease.

In an *in vivo* mouse model, FabC11:Stx2 protected mice from death after a lethal dose of Stx2. The non-protected intoxicated animals exhibited high urea concentrations after 72 h, suggesting that they died from kidney damage related to Stx2 cytotoxicity. Tests with separate inoculation of Stx2 and the antibody did not show protection (data not shown). The inability of Fab fragments to protect in an in vivo model without prior incubation with the target toxin was also shown by Akiyoshi et al. [24], with Fab and F(ab)_2_ against Stx2 B subunit, and by Mejías et al. [9] with VHH produced in camelids also against Stx2. This may be explained by the molecular weight cutoff of around 30–50 kDa for glomerular filtration [25]. Since the predicted size of FabC11:Stx2 is 48 kDa, this may be too close to the cutoff, resulting in a decreased half-life for this fragment in the circulation, where the antibody would be cleared from the animal’s blood before encountering the toxin. This could be improved by fusion to a long-lived serum protein such albumin as demonstrated by Mejías et al. [9], or by transforming the Fab fragment into a full-length IgG4 antibody by sub-cloning, which is currently being done by our group. The plasticity of the recombinant molecule makes it possible in an easier manner compared with monoclonal antibodies from hybridoma. 

In summary, the results obtained with FabC11:Stx2 showed that this Fab recombinant fragment was able to prevent Stx2 toxicity to human kidney cells and in mice. Thus, we conclude that FabC11:Stx2 is an encouraging therapeutic tool for neutralizing the effects of Stx2 and preventing HUS development, even though more investigations are needed regarding the efficacy of this protection in vivo.

## 4. Materials and Methods

### 4.1. Reagents

Purified Stx2a is commercially available and obtained from Phoenix Laboratory, Tufts Medical Center, Boston, MA, USA. Recombinant Stx2 (rStx2) was expressed and purified as previously described [26]. FabC11:Stx2 recombinant antibodies were produced as previously reported [14].

### 4.2. FabC11:Stx2 Affinity by Surface Plasmon Ressonance

The surface plasmon resonance was performed and analyzed using a BIAcore T200 instrument (GE Healthcare, Uppsala, Sweden), following the manufacturer’s recommendations. In summary, a censor chip (CM5) was prepared for immobilization of Stx2 or Stx1 (5 μg/mL) in 10 mM sodium acetate buffer, pH 5.5 (152 RU) and activated by *N*-ethyl-*N*-(dimethylaminopropyl) carbodiimide (EDC) and *N*-hydroxysuccinimide (NHS) in a mix of equal amounts. The HEPES-EDTA (HBS-EP buffer, pH 7.4, containing 10 mM HEPES, 150 mM NaCl, 3 mM EDTA, and 0.05% Tween 20 was used as running buffer and the samples were prepared in HBS-EP buffer (0–7.4 μM, twofold dilutions). A multicycle model held at 25 °C and a 30 μL/mL flow rate (120 s contact and 600 s dissociation) was performed for kinetic study. The sensor chip regeneration was performed between cycles using a 15 μL pulse of 100 mM glycine plus 2 M MgCl_2_, with a pH of 2. The experiments were performed in duplicate and using the Langmuir 1:1 binding model, the K*_D_* was calculated by BIAevaluation 3.0 (GE Healthcare, Uppsala, Sweden).

### 4.3. Primary Culture

Human glomerular endothelial cells (HGEC) were obtained from kidney fragments of different human pediatric patients, which were undergoing nephrectomy at Hospital Nacional “Alejandro Posadas,” Buenos Aires, Argentina. Patients exhibited either segmental uropathies or a tumor in one pole and normal values of creatinine. Fragments were taken from kidney normal areas. Written informed consent was obtained from the next of kin, caretakers, or guardians on behalf of the minors/children participating in our study. The study was conducted in accordance with the Declaration of Helsinki, and the protocol was approved by the Ethics Committee “Dr. Vicente Federico Del Giúdice” of the Hospital Nacional “Alejandro Posadas” (035LUP1So/17(13)). Experiments were developed in growth-arrested conditions by using a medium M199 with 10% fetal bovine serum (FBS), 3.2 mM L-glutamine and 100 U/mL penicillin/streptomycin (P/E), and without an endothelial cell growth supplement. Cells were used between 2–7 passages, after endothelial characterization, as was previously described [16].

### 4.4. Cell Line Culture

Human proximal tubular epithelial cell line (HK-2) was grown in Dulbecco’s Modified Eagle Medium (DMEM F12) (Sigma Aldrich, St. Louis, MO, USA) with 10% FBS, 1% P/E (GIBCO, Invitrogen, Carlsbad, CA, USA), 2 mM L-glutamine, 15 mM HEPES at 37 °C in a humidified 5% CO_2_ incubator. Experiments were developed in growth-arrested conditions with DMEM/F12 medium without FBS.

### 4.5. Stx2 Neutralization Assay in HGEC and HK-2

For neutralization assays, HGEC and HK-2 cells were treated using two different protocols. For pre-incubation assays, cells were pre-treated with different concentrations of FabC11:Stx2 (1 h at 37 °C) and then exposed to Stx2 for 72 h. For co-incubation assays, cells were treated simultaneously with FabC11:Stx2 and Stx2 for 72 h. FabC11:Stx2 concentrations used ranged from 0.001 to 10 µg/mL. Stx2 was assayed at the dilution required to kill 50% of cells (1 CD_50_ = 0.1 ng/mL). The molar ratios used ranged from 0.7:1 to 7000:1 for Fab:Stx2. Finally, cell viability was analyzed as is described below.

### 4.6. Neutral Red Cytotoxicity Assay

HGEC and HK-2 cell viability was analyzed by neutral red uptake assay, as was previously described [27]. HGEC and HK-2 cells were grown to confluence in 96-well plates with a complete medium. Treatments were developed for 72 h and then freshly diluted neutral red (Sigma Aldrich, St. Louis, MO, USA) was incorporated to a final concentration of 10 mg/mL. After that, cells were incubated for an additional 1 h at 37 °C in 5% CO_2_. Finally, cells were washed and fixed with 1% CaCl_2_/1% formaldehyde and then lysed with 1% acetic acid in 50% ethanol. Absorbance was measured in an automated plate spectrophotometer at 540 nm. Results were expressed as percentage of viability, where 100% represents cells incubated under identical conditions but without toxin treatment. Percentage of cell death prevention was calculated considering 100% protection when Stx2 cytotoxic effects were totally reversed.

### 4.7. Cell Morphology Analysis

HGEC and HK-2 cells were seeded on glass coverslips (12 mm), washed with PBS at pH 7.4 and treated as described above. For these experiments, 10 µg/mL FabC11:Stx2 was used. After incubation, cells were fixed for 2 h at room temperature with 96% *v*/*v* alcohol, stained with hematoxylin/eosin (H&E), and examined by light microscopy (×200 and ×400, Zeiss Axiophot, Zeiss, Heidelberg, Germany). To obtain the percentage of cells/field, photographs of 10 randomly selected fields were taken and then cells were counted and averaged. The percentage of cells per field was calculated considering the average number of control cells as 100% (percentage of cells/field = (number of treated cells × 100)/number of control cells). From the same photographs, the percentage of cell area was calculated; the cell area in each cell was analyzed using the ImageJ software (NIH) according to the manual instructions. The average of the cell areas was calculated in each condition and the control cell area was considered as 100% (percentage of cell area/field = (cell area of treated cells × 100)/cell area of control cells). Results are expressed as means ± SEM. Percentages of cell detachment and intracellular edema prevention were calculated considering 100% protection when these effects were totally reversed.

### 4.8. In Vivo Stx2-Neutralizing Capacity

BALB/c mice were bred in the animal facilities of the Instituto de Medicina Experimental (IMEX), Buenos Aires. The experiments performed in the present work were approved by the IMEX Care Committee (ethical approval code: CICUAL No. 34/17, date of approval: 24 May 2017) in accordance with the principles set forth in the Guide for the Care and Use of Laboratory Animals. Throughout the studies, the health and behavior of the mice were assessed three times a day. Any unnecessary pain, discomfort, or injury to animals was avoided. To test in vivo Stx2-neutralization activity and protective capacity of FabC11-anti-Stx2 antibody (FabC11:Stx2), rStx2 (one lethal dose of 100%, 1 LD_100_, 1 pmol/mouse) was pre-incubated with FabC11:Stx2 (2 μmol/mouse) or with PBS as negative control, for 1 h at 37 °C and 1 h at 4 °C. The mixture was injected intravenously into naive adult Balb/c mice and survival was checked. For each group of experiments, three mice were tested. Blood samples were obtained at 72 h for determination of blood urea nitrogen in plasma. Biochemical determinations of urea in mouse plasma were performed with a commercial kit (Wiener Lab, Buenos Aires, Argentina).

## Figures and Tables

**Table 1:** SPR of FabC11:Stx2 for affinity determination against both Stx toxins. The monoclonal antibody (mAb) Stx2 [15] was used as positive control. The values represent two independent Fab batches.

Sample	ka (1/Ms)	kd (1/s)	K*_D_* (M)
Fab:C11 vs. Stx1	(4.4 ± 0.9) × 10^5^	(1.5 ± 0.2) × 10^−2^	(3.4 ± 0.2) × 10^−8^
Fab:C11 vs. Stx2	(4.2 ± 0.2) × 10^4^	(2.9 ± 0.1) × 10^−4^	(7.0 ± 0.1) × 10^−9^
mAb Stx2	(1.0 ± 0.2) × 10^4^	(2.0 ± 0.3) × 10^−4^	(2.0 ± 0.1) × 10^−8^

ka, association rate constant; kd, dissociation rate constant; K*_D_*, kinetic affinity constant.
